# Enhancing fracture repair: cell-based approaches

**DOI:** 10.1097/OI9.0000000000000168

**Published:** 2022-03-10

**Authors:** John Wixted, Sravya Challa, Ara Nazarian

**Affiliations:** a Department of Orthopedic Surgery, Beth Israel Deaconess Medical Center; b Harvard Combined Orthopedic Residency Program, Boston, Massachusetts

**Keywords:** fracture, HIF, orthopaedic surgery, skeletal stem cell, WNT

## Abstract

Fracture repair is based both on the macrolevel modulation of fracture fragments and the subsequent cellular activity. Surgeons have also long recognized other influences on cellular behavior: the effect of the fracture or subsequent surgery on the available pool of cells present locally in the periosteum, the interrelated effects of fragment displacement, and construct stiffness on healing potential, patient pathophysiology and systemic disease conditions (such as diabetes), and external regulators of the skeletal repair (such as smoking or effect of medications). A wide variety of approaches have been applied to enhancing fracture repair by manipulation of cellular biology. Many of these approaches reflect our growing understanding of the cellular physiology that underlies skeletal regeneration. This review focuses on approaches to manipulating cell lineages, influencing paracrine and autocrine cell signaling, or applying other strategies to influence cell surface receptors and subsequent behavior. Scientists continue to evolve new approaches to pharmacologically enhancing the fracture repair process.

## 1 Introduction

Fracture repair is fundamentally a process of cell biology. Surgeons have, ostensibly through a decades-long collective effort of trial and error, developed a practical understanding of how the fracture-healing process is mechanically regulated. Our understanding of the process, indeed, is usually taught and discussed in terms of the macroscale biomechanical influences such as construct stiffness, which surgeons manipulate to attain optimal clinical outcomes.^[1,2]^ Like any physiologic process, however, the underlying biology of skeletal regeneration is a highly coordinated cellular process based on many cell types and signaling molecules.^[3]^ Surgery can actively manipulate the external environment of healing fractures; the subsequent changes are reflected through mechanotransduction at the cellular level.^[4]^ Surgeons have also long recognized other influences on cellular behavior, such as the effect of the fracture or subsequent surgery on the available pool of cells present locally in the periosteum, the interrelated effects of fragment displacement and construct stiffness on healing potential, patient pathophysiology and systemic disease conditions (such as diabetes), and external regulators of the skeletal repair (such as smoking or effect of medications).^[5]^

As our collective understanding of the cellular physiology of fracture repair has broadened, extensive efforts have been made to pharmacologically enhance fracture repair. These might take any number of approaches, from decreasing nonunion rates to shortening the time to achieve union of fractures, treated either with or without surgery. The common thread among these efforts is to manipulate the biology of cells involved in the fracture repair process to drive the desired changes. This review is by no means an exhaustive effort at enumerating the many directions these efforts have taken. Rather, the goal is to give a general understanding of how several translational approaches have approached the challenges of skeletal regeneration, and to illustrate how what we know of cell signaling and skeletal repair might be used to inform our efforts at improving patient care.

## 2 Approaches

### 2.1 Modifying cell lineage

Fractures fundamentally result in local tissue trauma, both osseous and nonosseous, and this includes disruption of the periosteum with local tissue hypoxia. This in turn leads to a cytokine-driven inflammatory response, which initiates a cascade of intra- and extra-cellular events, culminating in skeletal regeneration. Our understanding of that cellular response has evolved in recent years. Earlier studies on cell populations involved in the fracture-healing cascade identified groups of cells which demonstrated varying degrees of pluripotency, termed mesenchymal stem cells (MSCs). With more sophisticated techniques, including surface marker analysis and genetic labeling, researchers have been able to demonstrate that MSCs likely represent a heterogeneous cell population; while undifferentiated MSC cell populations can be identified in a host of tissues including adipose, muscle, and bone tissue, the pluripotency of these cells is variable.^[6,7]^ Specific cell populations termed Skeletal Stem Cells (SSCs), which are present in bone and periosteum but not in other tissues such as adipose, appear to better represent immature cell populations with the potential to differentiate into the chondroid and osteoid lineages that drive skeletal regeneration.^[8]^ After the identification of SSCs, further characterization has identified subpopulations within the SSC grouping, a trend that is likely to continue as our understanding of these cell types continues to grow. In response to injury, SSCs from the periosteum and/or the bone marrow compartment generate bone through a hybrid osteochondral intermediate or through direct ossification via osteoprogenitors^[9]^ In some contexts, such as the zebrafish fin and murine digit tip, osteoblasts can dedifferentiate and redifferentiate to produce new bone. Sometimes, bone healing involves cells that do not strictly qualify as stem cells. For example, some studies observed dedifferentiation of osteoblasts in zebrafish bone regeneration and murine digit tip regeneration.^[10,11]^

Various efforts at driving stem cell populations toward committed cell types, both chondroid and osteoid, have been studied for their ability to enhance fracture repair. Bone morphogenic proteins, acting primarily as a secreted growth factor, have long been identified as drivers of cell lineages toward both chondroid and osteoid lines.^[12]^ A wide range of identified bone morphogenic proteins (BMPs) act on cells within healing fractures, and the addition of recombinant human BMP-2 delivered via bovine collagen matrix has been used clinically to enhance fracture repair. The effects of BMPs are widespread, regulating differentiation of both chondroid and osteoid lineages, and the effects of various BMPs are highly contextual. The effects of BMPs on driving cell lineage and differentiation require cofactors to influence downstream effects on Runx-2, among other transcription factors.^[13]^

### 2.2 Autocrine and paracrine cell signaling

**Figure 1:**
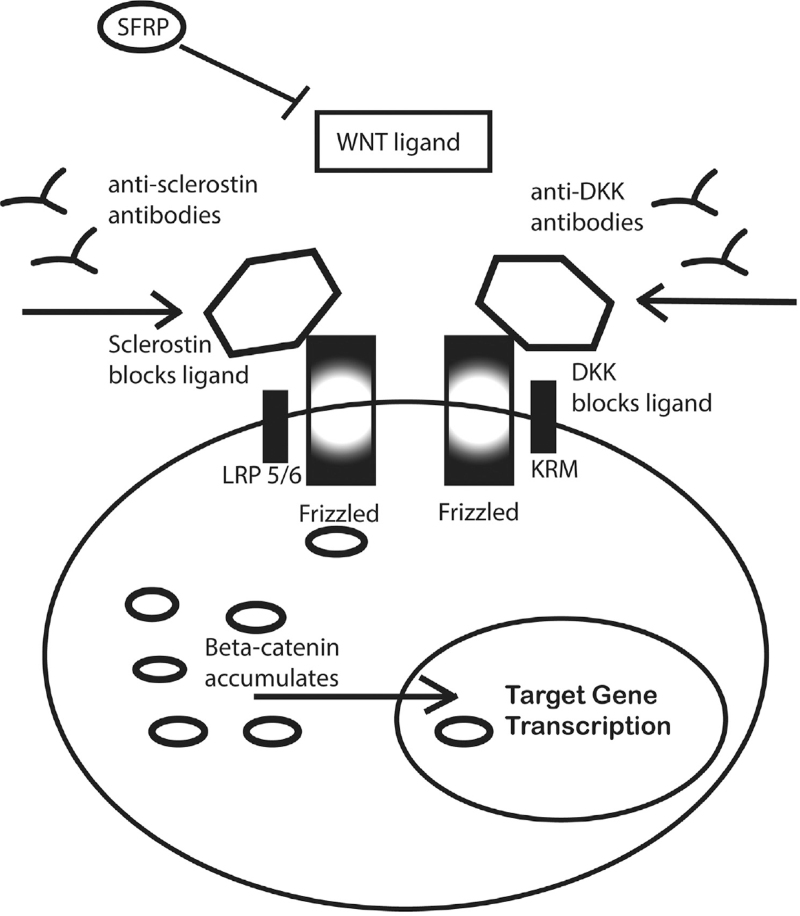
Simplified WNT signaling pathways. WNT ligands are blocked from binding Frizzled receptors and by sclerostin and DKK. Antibodies to sclerostin or DKK allow ligand binding. Frizzled complexes block beta catenin phosphorylation, leading to accumulation and translocation to the nucleus for activation of target gene transcription.

Modifying cellular behavior via small molecule effects on cell surface receptors is fundamental to pharmacology, leading to medications ranging from beta blockers to antihypertensives to cancer treatments. The Wnt pathways collectively are a group of cell signaling pathways that begin with activation of proteins on the cell surface, and allow for various cell–cell interactions or autocrine functions within the cell, eventually leading to downstream nuclear activity. Wnt signaling pathways, generally including canonical and noncannonical types, are well known to be crucial for embryonic development, as well as tissue-specific cellular differentiation and migration (Fig. 1). In the context of skeletal formation, Wnt signaling is critical for skeletal formation during embryogenesis, and this role is recapitulated during fracture repair. As the various Wnt signaling cascades are initiated at the cell surface, this makes them attractive targets for pharmacologic intervention. In a general sense, Wnt signaling is thus seen as a driver for fracture repair, and the inhibition of Wnt signaling has a negative effect on subsequent downstream cell behavior.^[14,15]^

Furthermore, a wide array of secreted molecules have been described which modulate Wnt signaling, among these are Secreted Frizzled Receptor Protein 1, sclerostin and DKK1 (Dickkopf WNT Signaling Pathway Inhibitor 1). Approaches that can affect cell surface receptors directly, or which target these various negative regulators for Wnt signaling, have been extensively studied for their effects on enhancing fracture repair. Small-molecule inhibitors of Secreted Frizzled Receptor Protein 1 have been demonstrated which enhance fracture repair.^[16]^ DKK1 antibodies that block the inhibitory effect of DKK1 on Wnt singaling have also been reported.^[17]^

However, demonstrating clinical effectiveness of these approaches has proven elusive. Romosozumab is an FDA-approved antisclerostin antibody therapy indicated for use in the treatment of osteoporosis. Given the well-demonstrated effect of such antibodies on preclinical models,^[18]^ investigators recently reported on 2 studies using romosozumab to enhance fracture healing in the tibia and the hip. Unfortunately, neither study was able to demonstrate efficacy in clinical practice. Romosozumab was tested in acute diaphyseal tibia fractures, with the primary outcome of radiographic healing failing to demonstrate efficacy of the treatment.^[19]^ Similarly, treatment with romosozumab was used as adjuvant in the treatment of hip fractures, and the primary endpoint of timed up-and-go testing, as well as patient-reported outcomes and radiographic union, failed to demonstrate a benefit to romosozumab treatment.^[20]^

### 2.3 Effect of hypoxia on chondrocyte behavior

**Figure 2:**
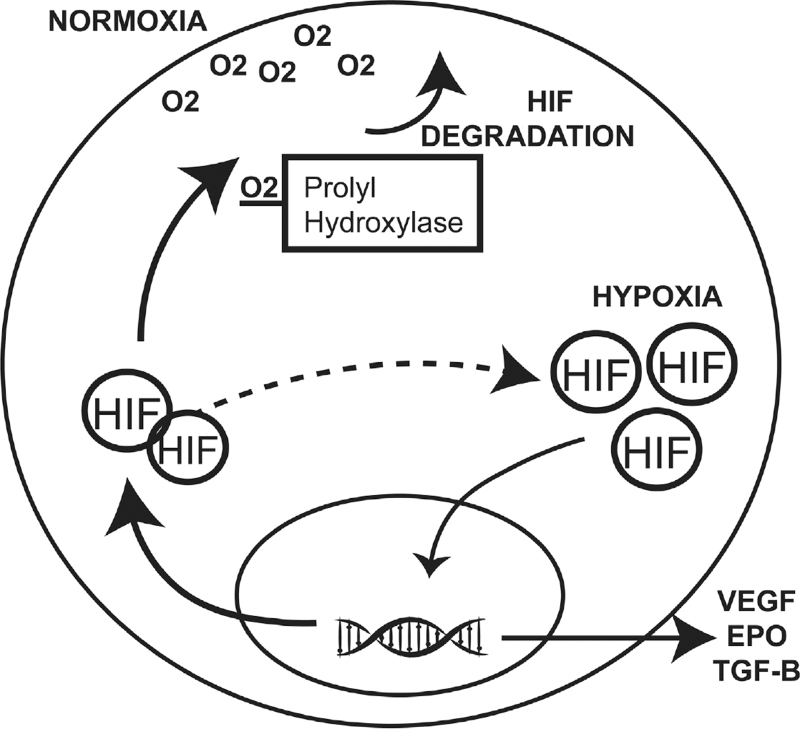
HIF signaling—normoxia and hypoxia. Under conditions of normoxia, HIF is degraded by an O_2_-dependent prolyl hydroxylase. In the absence of O_2_, HIF accumulates and translocates to the nucleus, activating transcription of HIF target genes. The prolyl hydroxylase can be blocked by a variety of pharmacologic agents, causing HIF accumulation.

One of the seminal events after fracture which drives the initial response to regeneration is local tissue hypoxia. After fracture, local Oxygen (O_2_) tension diminishes rapidly. Low O_2_ tension has been implicated as a driver for chondrocyte formation, and the Hypoxic Inducible Factor (HIF) pathways have been studied extensively for their effect on the early phases of fracture repair.^[21]^ While cell signaling approaches such as those described above may drive lineage changes within the cells, modifying hypoxia signaling is more akin to modifications of the external environment. Cells respond rapidly to changes in O_2_ tension via HIF signaling. Cells within the fracture callous will produce HIF, and this intracellular protein is rapidly broken down by the prolyl hydroxylase enzyme (PHD), which requires O_2_ as a co-factor. In the absence of O_2_, the PHD is ineffective, and HIF accumulates rapidly. This intracellular accumulation of HIF leads to nuclear translocation and subsequent vascular endothelial growth factor (VEGF) production, among other effects (Fig. 2).

Small molecule approaches which block PHD in an O_2_ independent fashion have been tested in animal models and shown to dramatically increase neo-angiogenesis, leading to large and rapid callous formation at the fracture site. By blocking the enzymatic effect of PHD, HIF cannot be broken down, and in turn drives an increased cellular response to perceived hypoxia. Animal models using deferoxamine in a variety of settings have consistently demonstrated its positive effect on fracture repair, highlighting HIF modulation as a potential means of accelerating fracture healing.^[22]^

Clinically, a number of orally administered, small molecule HIF prolyl hydroxylase inhibitors are in trials for treatment of anemia. Roxadustat, for example, is approved for use in China and the European Union as a treatment for renal induced chronic anemia.^[23]^ As with anti-sclerostin antibody treatment, ample preclinical evidence in animal models would suggest that prolyl-hydroxylase inhibitors might have positive effects on human fracture treatment.^[24]^ Currently, Roxadustat has not been approved by the FDA for treatment of anemia in chronic kidney disease due to concerns over its safety profile. Furthermore, experience with Romosozumab also provides a cautionary tale about the need for robust clinical trials of any approach to healing fractures prior to adoption. Other oral, small molecule prolyl-hydroxylase inhibitors are also in trials pending FDA evaluation, including Vadadustat^[25]^ and Daprodustat,^[26]^ both seeking approval for the treatment of anemia associated with chronic kidney disease.

### 2.4 Influencing mechanotransduction

One primary effect of surgical treatment for fracture repair has been modulation of the mechanical environment, and surgeons have long understood in general terms how fractured bone behaves under various conditions of loading and stability. Recent evidence regarding chondrocytes, critical for endochondral ossification, has implicated mechanically regulated calcium channels in mechanotransduction.^[27]^ Conformational changes to the chondrocyte, initiated by external loading, leads to activation of these channels, and a growing understanding of the channelsome in cartilage cells opens a new range of possibilities for enhancing fracture repair.^[28]^ Stretch-evoked calcium channels are now recognized as responding to tissue strain. This may have significant implications for both arthritis and for fracture repair. Recent reports indicate a role for transient receptor potential vanilloid -4 in responding to strain and mutations in transient receptor potential vanilloid -4 have been associated with skeletal dysplasias.^[29]^ The role of calcium dependent mechanotransduction during fracture repair is not yet elucidated.

On a macro scale, however, mechanical stimulation of healing fractures via ultrasound treatment and by electromagnetic stimulation has an extensive history in clinical practice. More recent and robust studies in tibia fractures, however, have brought into question the efficacy of low intensity pulsed ultrasound as a means of enhancing fracture repair.^[30]^

### 2.5 Immunomodulation

Fracture repair is inherently an inflammatory mediated process. As such, it stands to reason that significant overlap would exist between the immune system and the processes which drive both endochondral and intramembranous bone formation. Clinicians have long recognized the deleterious effects on fracture repair of broad, systemically administered immune suppressants such as prednisone and methotrexate. The immune system presents both a scientific and a practical challenge, as the scale and breadth of immune system cell types and potential modulators is enormous. With the vast array of both pro- and anti-inflammatory cytokines influencing various immune and osseous cell behaviors, finding specific mediators that might be used to enhance fracture repair without causing unintended consequences is difficult.^[31]^ Our collective understanding of the interplay between macrophage cell subtypes, t-cells, other immune cell types, and their respective cytokines with healing fracture cells is incomplete at best.

Broadly, macrophages are widely recognized as being present during both early and late phases of fracture repair, and have been studied for their role during endochondral ossification. The M1 and M2 phenotypes of macrophages appear to influence fracture repair differently, and the ratio of M1/M2 macrophages present during fracture healing has been studied with knock-out mouse models, demonstrating that enhancement of M2 macrophages can help drive osseous tissue formation late in the process.^[32]^ Given this, various biomaterials that can themselves modulate macrophage transformation are also under investigation, leading to an entirely different approach to enhancing fracture repair. Rather than pharmacologic intervention, this approach would influence the local immune response via engineered materials designed to modulate local inflammatory mediators.^[33]^ While in its infancy, such approaches would take a local, rather than a systemic, approach to mediating fracture repair.

## 3 Conclusion

In summary, a wide variety of approaches have been applied to enhancing fracture repair by manipulation of cellular biology. Many of these approaches reflect our growing understanding of the cellular physiology that underlies skeletal regeneration. By manipulating cell lineages, influencing paracrine and autocrine cell signaling, or applying other strategies to influence cell surface receptors and subsequent behavior, scientists continue to evolve new approaches to pharmacologically enhancing this process. These have not been met with a great deal of translational success, seemingly a reflection of both the robust nature of fracture repair and the limitations of our ability to quantify the healing process. New approaches to influencing this process will continue to evolve as our understanding of the mechanics and cellular interactions evolve.
